# A qualitative assessment of implementing a cross-cultural survey on cancer wards in Denmark - a description of barriers

**DOI:** 10.1186/1471-2288-10-4

**Published:** 2010-01-12

**Authors:** Maria Kristiansen, Amani Hassani, Allan Krasnik

**Affiliations:** 1 Section for Health Services Research, Department of Public Health, University of Copenhagen, Copenhagen, Denmark

## Abstract

**Background:**

Research into migration and health is often confronted with methodological challenges related to the identification of migrants in various settings. Furthermore, it is often difficult to reach an acceptable level of participation among migrant groups in quantitative research. The aim of this study is to conduct a qualitative assessment of the barriers encountered during the implementation of a cross-cultural survey on cancer wards in Copenhagen, Denmark.

**Methods:**

Participant observation at the involved wards was combined with qualitative interviews with selected nurses and informal talks with a wider group of nurses at the wards involved in the survey.

**Results:**

One possible way to increase the participation of migrant patients in research is through the involvement of the hospital staff in contact with patients. Involvement of nurses on cancer wards in the delivery of questionnaires to patients was challenging, despite a general willingness to participate in psychosocial research. The main difficulties were found to be both external (policy changes, general strike among nurses) and internal on the wards (heavy workload, lack of time, focus on medical aspects of cancer rather than psychosocial aspects). These factors interacted and resulted in a lower priority being given to psychosocial research. Further, nurses expressed a feeling that researchers in general did not recognize their contribution in research, making it more difficult to engage fully in studies.

**Conclusions:**

Involving hospital staff in research is feasible but not straightforward. Awareness of the influence of possible external and internal factors and efforts to deal with these factors are fundamental to the successful implementation of psychosocial cancer research in a hospital setting.

## Background

Studying disease patterns and access to health services among migrant populations has become increasingly important given the growing number of immigrants and their descendants in Europe. A rising number of studies point to inequalities in morbidity and mortality for diseases such as diabetes, heart disease, psychotic disorders and cancer [1-3]. Furthermore, there is documentation of inequality in access to health services among migrants compared with the background population in various European countries [1,4-6]. However, little is known about access to social resources, such as social support, and how this relates to factors such as migrant status, social position, gender and age.

Research in the field of migration and health is a developing area with on-going discussions concerning the methodological, conceptual and theoretical challenges facing researchers. Apart from the controversy surrounding the terminology, assignment and classifications of ethnicity in various populations and for various purposes, much work remains before conclusions can be drawn about the reasons for the differences in disease patterns, mortality and access to health services [7-11]. A major obstacle is the difficulty related to collecting sufficiently detailed data for a sufficient number of migrants in order to understand why these discrepancies exist [12]. Data for a substantial number of migrants are required to ensure that findings are applicable to diverse populations representing the demographic composition of a given country and to make it possible to do ethnicity-specific analyses, thereby avoiding crude analyses disregarding the large heterogeneity among migrant groups [12]. Register studies have the potential disadvantage of lacking relevant and validated data on migrant status/ethnicity, resulting in inaccurate and incomplete data [13]. Furthermore, studies relying on self-assignment of migrant status/ethnicity (measured as e.g. birthplace, nationality or self-perception of ethnic identity) may encounter difficulties in reaching a satisfactory level of participation among migrant groups, again causing data to be incomplete [7]. Although postal questionnaires may reach large population samples and be easy to administer, a Swedish study found that non-participation was common among migrants, thus reducing the external validity of the survey and making robust conclusions about ethnic differences difficult [14]. Other studies using postal survey methodologies or telephone interviews have found increased odds for non-response related to ethnic minority status as well as other characteristics such as degree of urbanization and low educational background [15-18]. The non-response may be due to factors related to migrant status, such as language difficulties, low literacy levels, low educational level, lack of familiarity with and distrust of research, and seriousness of disease status, causing migrants to decline participation [14,19-21]. For example, a recent British study reflected on the use of questionnaires to evaluate patient experiences and outcomes among diabetes patients of Bangladeshi origin [22]. The study encountered profound difficulties in administering questionnaires to this ethnic group, who found the structure and design of questionnaires difficult to comprehend and complete [22]. However, low response rates may also be due to inadequacies in the methodology used, e.g. too complicated and poorly translated questionnaires [21].

This study was initiated as a consequence of methodological problems related to a cross-cultural survey involving cancer wards at three large hospitals in Copenhagen 2007-2009. The survey was part of a larger study using both qualitative and quantitative methods to explore differences and similarities in needs for and experiences with social support among Danish-born and migrant cancer patients originating from seven non-Western countries. Given the difficulties in recruiting migrants into research projects, we recruited from care settings through personal contacts between patients and hospital staff. This has been found to facilitate participation rates in other studies [12]. After agreement with the management of cancer units, nurses acted as go-betweens and gave the questionnaires to patients. This was decided upon because of the close contact nurses have with patients and nurses' focus on psychosocial adjustment to cancer - these factors were perceived to be less prevalent among doctors. Nurses on the wards involved were informed about the study at a conference for cancer nurses and at morning ward meetings. During these meetings any difficulties or questions were addressed with the aim of supporting nurses and motivating them to give questionnaires to patients. Furthermore, nurses were offered seminars about the background for the survey as well as the preliminary results of the overall study in order to motivate them to engage in the survey. Further meetings were held throughout the duration of the survey. Finally, the participating wards were given gifts to show our appreciation of their involvement. In spite of these efforts, however, the response rate was very low mainly due to insufficient distribution of questionnaires to patients by the nurses. In order to provide a better basis for involvement of nurses in future studies of this kind we decided to carry out an additional methodological study on determinants of the low participation of nurses.

The aim of this paper is to present the results of a qualitative assessment of the barriers encountered during the implementation of this cross-cultural survey on cancer wards in Denmark.

## Methods

### Participant observation

During the planning and implementation of the questionnaire study, MK and AH did participant observation and made field notes based on observations and informal talks with nurses on the wards, mainly during identification of patients and when placing questionnaires in patients' files. Additionally, morning meetings were held for nurses working on the different wards to inform them about the study and motivate them to hand out questionnaires, and to gain insight into their perceptions of the study and their experiences with handing out questionnaires to patients. These meetings were not scripted but rather conducted with a clearly specified verbal presentation of the project's objectives. Either MH or AH attended these meetings and subsequently compared field notes about the nurses' motivation and experiences. During these encounters issues arose concerning attitudes and motivation to participate in research within the field of psychosocial research among migrant cancer patients, the barriers experienced in participating in this kind of research, as well as issues concerning needs and advice in relation to the research project.

### Semi-structured interviews

To validate and further clarify our observations in the different wards, semi-structured interviews were later conducted by one of the authors with a head nurse (age: 33 years, seniority: 7 years) on a lung cancer ward, and a clinical nursing supervisor (age: 51, seniority: 24 years) and a staff nurse (age: 59, seniority: 33 years) both working on a ward treating patients with cancer of the head and neck. The different categories of nurses were chosen to represent different hierarchal as well as different seniority levels and thus gave a diverse perspective and explanation of the experienced barriers. The staff nurse and the clinical nursing supervisor were identified by the head nurse in the head and neck cancer ward, while the head nurse at the lung cancer ward was asked directly. These two wards were chosen because they were perceived to represent diversity in relation to type of patient population, motivation and possibility for participating in the research project.

Semi-structured interviews allowed us to conduct a further and more in-depth qualitative assessment of the nurses' experiences noticed and noted in the field notes. Semi-structured interview was chosen as a method since it is an open and dynamic way of conducting interviews on the basis of a general interview guide with the main themes outlined, however with a flexibility of further questioning the answers given by the nurse during the interview. Based on a semi-structured interview guide the following themes were discussed during interviews: attitudes to and motivation for nurses' involvement in psychosocial cancer research, barriers experienced to giving questionnaires to patients and their possible interrelatedness to characteristics among specific subgroups among cancer patients (migrants and patients with lower social position), and finally advice to researchers planning to involve nurses in cancer research. Interviewing nurses enabled us to ground barriers and recommendations in the organisational practice embracing encounters between nurses and patients.

The survey study was approved by the Danish Data Protection Agency which is a national agency securing storage and use of information about individuals. Furthermore, the study was granted approval by the management level at the involved hospitals and by heads of the individual wards. No approval from ethical committees is needed for interview studies according to official Danish research guidelines. However, the study is in compliance with the ethical principles for medical research as presented in the Helsinki Declaration. Interview persons were introduced to the study orally and were adequately informed of the purpose and methods of the study. Furthermore, issues concerning anonymity and the right to withdraw from the study at any time were described. Informed consent was given orally.

### Data analysis

Interviews were voice recorded and subsequently transcribed verbatim. Data was analysed thematically by the authors who conducted the analysis first independently, and later compared and discussed initial interpretations which were then adjusted. During the process of interviewing patients and analyzing data constant attention were given to securing the credibility of results. This was done by being reflexive, first to both interviewing style by reading field notes and listening to recordings of interviews followed by adjusting and refining questions between interviews, and secondly to analysis by presenting interim themes to succeeding interview persons, and by questioning and discussing immediate interpretations of data within the research group as well as at seminars including a wider group of cancer researchers from different academic traditions.

## Results

Despite the nurses' general willingness to participate in the project, we found both external and internal factors that influenced nurses' engagement in the distribution of questionnaires to cancer patients, regardless of whether patients were Danish-born or migrant. The external factors are defined as factors arising outside the particular hospital ward, whereas internal factors originate within the respective wards. As will be apparent in the following, these factors are highly interdependent. However, we have chosen to describe them separately in order to illustrate clearly the specific challenges experienced within the cancer wards. The following results are based on field notes taken during participant observation and informal talks with nurses as well as interview data.

### Nurses' willingness to assist in the survey

Initially, when informing nurses about the study on the different hospital wards, the authors received positive feedback and encountered a general willingness to participate. As one nurse on a head and neck cancer ward expressed:

"...It is really relevant to be part of research when we are working with people. Especially within this field of research."

However, there was a difference in the degree of willingness to participate across the different wards. For instance, one head nurse of an oncology ward treating lung cancer patients emphasized the specific importance of psychosocial research for their patient population of critically ill patients:

"They are seriously ill when they get here. Survival [in this ward] is only 8%, so I think our starting point is a bit different from other wards. We know how to step it up from the beginning [and] we know it's the small things on the personal level that make a difference."

Another head nurse from a different oncology ward explained that due to the high level of stress on the ward, it was necessary for nurses to prioritise, and while the issue of social support was very important, the issue of the correct medical treatment could have greater consequences for the individual patient and thus took precedence over psychosocial research. During informal talks with nurses it was mentioned that for patients with lung cancer, treatment possibilities and subsequently chances of survival are often limited, which makes psychosocial support related to quality of life important.

A further difference in willingness to assist in the survey is related to the differing priorities and possibilities that exist for nurses at the management level and the staff level respectively. Many researchers want to involve staff and cancer patients in research projects with differing aims and methods and this creates a situation for head nurses where prioritizing becomes paramount. As a head nurse explained:

"For me as a head nurse, I have to prioritize. I get a lot of enquiries all the time, like "can't we just do this..." and I'm sort of "yes, of course we can. It is so exiting". But at the same time, I feel that there is a responsibility on the part of those who feel like doing a project...I would like to join in and I think it is exiting to be part of it and all. But all this work keeping check on who wants to hand out stuff and all that. I just can't do this.

As a consequence, during meetings with nurses at management and staff level it was often emphasized that it was critical for researchers and management to get the acceptance and involvement of staff nurses, since much work rested on nurses at the staff level.

Thus, despite the general willingness to engage in psychosocial research amongst the nurses, we found differences in motivation and possibilities to participate in the study depending on type of cancer being treated and the status in the hospital hierarchy.

### External factors affecting the survey

During the course of the study several changes introduced by the external environment had a marked, limiting effect on nurses' possibility to participate in the study. Political decisions introducing treatment guarantees within certain time limits for cancer patients at Danish hospitals caused an increased focus on the medical aspects of treatment. This combined with massive media coverage of system failures to live up to the guarantee, necessitated nurses de-prioritizing participation in psychosocial research. As one nurse explained:

" [There are] so many externally disturbing elements that you have to be preoccupied with [...] giving the treatment, and then there is the difficulty of the treatment getting delayed. Other things simply take over and become the centre of attention, not necessarily because we want them to, but because we are a part of giving the treatments."

Furthermore, a major strike amongst nurses in the spring of 2008 led to no distribution of questionnaires in a period of 4 months followed by a period of slowly uptake of the delivery of questionnaires due to heavy workloads after nurses went back to work. Union guidelines prohibited nurses from involving in activities not essential for the treatment of cancer patients during the strike, which obviously included questionnaire surveys. It is hard to quantify these external impacts upon the survey in terms of number of questionnaires delivered, but during participant observation it became clear that the survey was terminated completely during the strike and was only slowly resumed.

One nurse explained how external factors (e.g. the treatment guarantee and the strike) had created such a tremendous work pressure on the wards that it had influenced how much time the nurses were able to spend with the patients:

"Time shouldn't explain everything, but I think that some external factors have made it [the job] so busy that time has become a factor... [when] we have so little time for each patient, then it becomes a factor that counts."

Thus, external factors that were unanticipated at the time of planning the survey played a major role in limiting nurses' ability to engage in the survey, despite their general willingness to participate.

### Internal factors affecting the survey

Whilst the external factors were mainly disruptive elements from outside the hospital wards, we also experienced internal challenges affecting the nurses' involvement.

#### Influence of high staff turnover

Several wards participating in the survey experienced a high staff turnover resulting in a heavier workload among remaining staff. The existence and impact of this high staff turnover was evident both during participant observations and informal talks. One head nurse explained how this had had an effect on research:

"With a restricted number of staff and many new [nurses], we end up losing perspective about what goes on in the ward [...]. Things like this [research] become peripheral."

This meant further workload and stress for nurses, which resulted in a lack of over-view of the different types of research going on in the ward and a limited distribution of questionnaires.

#### Influence of workload and time

Generally, the workload for many nurses was overwhelming. Medical doctors were, in general, perceived to lack an interest in assisting in externally initiated research, which placed this responsibility on the nurses. As one nurse explained, it was a source of frustration among some nurses, who felt an obligation to assist in these studies but who, because of the workload, lacked the time and energy necessary. Subsequently, the time factor played a vital role in the nurses' capacity to assist in the survey. During morning meetings other nurses supported this opinion and explained how lack of time with the individual patient made it difficult to hand out the questionnaires. The fact that the patient needed a vast amount of information about his/her treatment during the short consultation with the nurse meant that time became a factor that resulted in de-emphasizing non-medical research.

Several nurses we interviewed and talked to informally expressed a feeling of being compelled to prioritize because of the overwhelming workload and limited time. In these instances, psychosocial research were the easiest to down-prioritize, whilst giving the correct treatment and conducting medical research were naturally always a priority, because of the possible consequences these factors could have on a patient's health.

#### Influence of medical priorities

The hospital as an institution focuses on providing the correct medical treatment. Thus, nurses talked about a pressure to prioritize the medical aspects of treatment over the psychosocial aspects, despite the many psychosocial aspects inherent in the nursing profession, as one nurse explains in the following:

"A project like yours [with a psychosocial focus] will come in second because you are in [this] area. As a nurse you are raised in the purely medical tradition."

The institution that the nurses are a part of thus becomes an important factor that can influence the nurses' approach towards psychosocial research. The focus on medical aspects of care created difficulties when approaching patients with more psychosocial issues. For example, one nurse explained during a morning meeting how she recognised the fact that they were assigned to hand out the questionnaires, but at times she found it difficult to introduce the subject to some patients. This insecurity towards approaching psychosocial issues could make it difficult to give the questionnaire to patients.

Nurses in general have the main responsibility of distributing material of different kinds to patients. In stressful periods when consultation time is scarce, nurses can feel forced to discriminate between the types of material to distribute. Due to limited time and the prevailing medical focus at the hospital, in some situations, nurses may prioritize material concerning medical treatment and research whilst deeming psychosocial research as too much for the patient to handle, as one nurse explained it. Although most nurses in this study expressed the need at times to discriminate between the materials distributed, there was a difference, depending on the different wards, related to the type of cancer being treated on the respective wards. Again, focus on the ward treating cancer of the head and neck was on medical treatment and medical research, whilst the ward treating lung cancer found the psychosocial aspect of treatment and research equally important. However, the prevailing focus on medical aspects of cancer care rather than psychosocial aspects influenced nurses' ability to engage in the study.

### Importance of recognizing nurses' contribution

An interview with the head nurse of a lung cancer ward gave some interesting perspectives as to which methodological approaches could influence nurses' willingness to participate in a particular research project. The head nurse explained how researchers sometimes take nurses' assistance for granted. A lack of recognition of the nurses' contribution and their general workload can undermine their efforts in assisting in a particular study. This experience was also expressed by several nurses during informal talks. The nurse explained how this makes the presentation of studies particularly important, since it is in the initial meeting with the particular wards that the researcher has the opportunity to express a degree of humility and gratefulness for the nurses' willingness to participate in the study. She emphasizes:

"You [as a researcher] will get far by showing respect for the nurses' work [...] It's about recognizing the nurses."

The head nurse further explained how greater personal contact with the nurses and greater insight into and presence in the particular wards can result in further knowledge of barriers and possibilities within the given ward, leading to increased respect for the nurses' contribution. This can give the researcher a better understanding of how to conduct the study in the most effective way; additionally, the researcher's mere recognition of the nurses' general work can increase the nurses' willingness to the participate in the study.

## Discussion

The main reason for including nurses in the delivery of questionnaires to patients was the assumption that during their contact with patients, nurses may facilitate participation in the study, as opposed to more passive recruitment strategies such as postal surveys. The focus on migrant patients, who are often socioeconomically disadvantaged and more difficult to include in studies, made this approach reasonable. Furthermore, migrant cancer patients are relatively few, making active recruitment strategies even more important. However, a prerequisite for the success of the chosen recruitment strategy was the active participation of nurses in delivering questionnaires to patients. In the course of the survey we experienced some barriers to nurses' involvement in psychosocial research. The barriers originated both in and outside the respective wards and interacted in a way that made it difficult for nurses to participate, despite their generally positive attitude towards psychosocial research. Two main external barriers had a substantial effect on nurses' participation in the survey. These barriers were the newly implemented treatment guarantee and a widespread strike among the nursing staff in the spring of 2008. Such external barriers were impossible to predict when planning the survey; however, they greatly limited nurses' ability to engage in the survey. In addition to the external barriers, we observed several internal barriers caused by different aspects intrinsic to the hospital ward. These barriers were generally connected to the everyday stressors in the ward, mainly heavy workload and lack of time, and the prevailing medical focus of the hospital. Such barriers made it necessary to give a lower priority to psychosocial research. Furthermore, nurses expressed a feeling that researchers in general did not recognize the contribution of nurses in research.

Professionals having to cope with huge workloads, changing policies setting new standards for their daily work and inadequate resources is a problem well documented [23,24]. In such situations the individual nurse must deal with dilemmas, trying to reconcile own preferences concerning nursing with organisational demands related to, for example, workload, time pressure and hierarchical structures [23,24]. The importance of psychosocial needs among cancer patients is increasingly recognized and is, furthermore, an essential part of the nursing profession. The importance of psychosocial research, and particularly the focus on vulnerable patient groups such as migrant cancer patients, was widely recognized among the nurses involved in the study. However, barriers made it difficult to engage in research of this kind.

For example, a tendency has been documented for staff to "cherry pick" patients who were considered approachable due to being of the same sex, social class and able to speak English fluently. While South Asians' attitudes to participation in trials were largely similar to the attitudes among the general UK population, exclusion from trials due to increased cost, time and language barriers combined with so-called passive exclusion due to cultural stereotypes among professionals led to underrepresentation of South Asians in trials [24]. It could be argued that nurses' prioritizing which material, including questionnaires, to give to the patient is a way of protecting the interests of the patient seen in the context of his/her disease, resources and vulnerability. However, it is possible that cultural stereotypes relating to which patients are able to complete a questionnaire can result in selective filtration of patients [24]. As shown, some nurses found it difficult to hand out the questionnaire to patients due to the sensitivity of the subject under study. This may result in a risk of selection bias due to staff selecting specific groups of patients to be given the questionnaire. Language barriers during encounters between nurses and patients may very well be a part of the reason for the difficulties in giving patients the questionnaire, but this was not mentioned during interviews. Difficulties in communication relate both to linguistic competence on behalf of the two parties involved and to cultural competency among nurses [24]. Nurses may feel overwhelmed by the task of involving patients with a migrant background due to inability to cope with language needs and cultural differences (such as the fear that giving the questionnaire may offend the migrant patient). However, barriers related to language and cultural differences were not identified during this study.

Involving the target groups, for instance by a dialogue with the target group when developing recruitment strategies, is important for the success of recruitment [20,25]. In our study we found early involvement of nurses and recognition of their contribution to research to be important factors, facilitating a fruitful corporation between nurses and researchers. Had financial incentives been offered to the nurses in addition to the other types of incentives provided (seminars and gifts) it would probably have increased the data collection rates. However, this was not possible due to financial restrictions on the survey. Conducting feasibility studies before the main study, i.e. by doing fieldwork on different wards, could have given important insight into some of the main barriers such as time pressure, workload and the culture of the institution.

### Methodological issues related to the assessment of barriers

The identified barriers to nurses' involvement in psychosocial cancer research are grounded in the experiences of the nurses interviewed as well as in observations and informal talks with other nurses working in cancer wards at a large Danish hospital. During these encounters with nurses we felt a willingness and openness to share both positive and negative perceptions and experiences. Being more than one researcher engaging with the nurses and thereafter comparing field notes gave us an opportunity to observe patterns that we probably would not have noticed otherwise. The interview data contributed to these observations and enabled us to further assess the strengths and weaknesses of the quantitative study design and the subsequent barriers experienced while conducting the study. However, if a third party not involved in the particular survey had collected the interview data, field notes and observations, it may have resulted in additional information that otherwise might have been withheld by the nurses or might have been beyond the researchers' realm of attention.

The head nurses of the departments were responsible for the selection of nurses for our interviews. This might create a bias if these nurses were chosen on the basis of special characteristics related to our research theme. It is very likely that the interviewed nurses as well as the nurses who participated in informal talks during the study had a more favourable attitude towards the study compared to nurses who declined to participate in interviews. This may influence the results of the qualitative assessment. However, other factors may prevent nurses from participating, e.g. lack of time or not being present at the day of interviews or informal talks. Our overall impression was that the same factors that prevented nurses from participating in the survey naturally also prevented them from participating in interviews and informal talks during the qualitative assessment, making it difficult to include a substantial number of nurses representing a wider section of the staff.

Other studies have found that barriers to inclusion of ethnic minorities in clinical trials may be related both to patient-factors, such as mistrust of the medical care system and of academic institutions, and to staff-factors, such as scepticism about the capability of low-income ethnic minorities to participate in research, lack of time and resources to inform patients about research and poor communication between staff and patients [11,12,19,24-26]. In this study we did not include patients' views on barriers to inclusion. Combining nurses' viewpoints with patients' experiences could have been beneficial. A qualitative British study on South Asian participation in clinical trials combined these two viewpoints, which resulted in identifying barriers difficult to identify when looking only at the staff's viewpoint [24].

The documented barriers are situated in a specific context related to the disease at hand (cancer), the type of research (psychosocial) and the chosen method (questionnaire study), whereas the focus on migrant patients as one of the target groups in the survey did not seem to play a significant role. While the psychosocial nature of the research clearly did play a role, findings may, nevertheless, be applicable to other types of research involving nurses within other fields of diseases. This may be due to the common internal barriers within hospitals settings, such as heavy workload and lack of time among nurses combined with a feeling of not being recognized. However, knowledge of specific barriers grounded in the local context is of vital importance to future research aiming at involving hospital staff in including patients into research.

## Conclusions

Inclusion of cancer patients through the involvement of health care professionals is feasible but challenging. Resources related to time and money must not be underestimated, because reaching patients through care settings and via involvement of nurses is costly and difficult to initiate. Gaining insight into external and internal barriers that make it difficult for nurses to participate in research is important at an early stage of planning research. Measures must be taken to overcome as many barriers as possible within the frame of a specific research project.

## Competing interests

The authors declare that they have no competing interests.

## Authors' contributions

The study was designed by MK and AK. Data were collected by MK and AH. Analysis, interpretation and write up were initially undertaken by MK and AH. Revisions and modifications were done by all three authors. All authors have given final approval of the submitted manuscript.

## Pre-publication history

The pre-publication history for this paper can be accessed here:

http://www.biomedcentral.com/1471-2288/10/4/prepub
